# Os Acromiale: Current Concepts in Anatomy, Diagnosis, and Management

**DOI:** 10.7759/cureus.107011

**Published:** 2026-04-14

**Authors:** Konstantinos Giatroudakis, Giannis Kotsalis

**Affiliations:** 1 1st Orthopedic Department, Athens General Hospital "G. Gennimatas", Athens, GRC

**Keywords:** acromial ossification centers, os acromiale, shoulder joint pain, subacromial impingement, surgical fixation

## Abstract

Os acromiale is a developmental anomaly resulting from the failure of fusion of acromial secondary ossification centers. Although often an incidental finding, it may occasionally be associated with shoulder pain, functional limitation, and other shoulder pathologies. Despite its relatively common occurrence in the general population, the clinical significance and optimal management of os acromiale remain subjects of ongoing debate. The acromion develops from the pre-, meso-, and meta-acromion centers, which typically fuse during adolescence or early adulthood. Failure of fusion most commonly occurs between the meso-acromion and meta-acromion, with reported prevalence varying across studies. Diagnosis is primarily clinical and radiological, with the axillary lateral radiograph being the most sensitive initial tool. Magnetic resonance imaging and single-photon emission computed tomography (CT), when combined with CT, are reserved for assessing fragment stability and associated soft-tissue pathology. Initial management typically starts with a conservative treatment period of variable duration before considering further interventions. Surgical treatment may be considered in patients with persistent symptoms despite prolonged conservative therapy, with fragment excision, acromioplasty, or internal fixation representing the most widely studied techniques. However, no single gold-standard technique has been established. Os acromiale is a common anatomical variant whose direct contribution to shoulder dysfunction is not always clear. Diagnosis requires a meticulous "exclusion-based" approach to ensure the fragment is the true pain generator. While conservative treatment remains the first-line approach, surgical intervention can provide satisfactory outcomes in selected symptomatic cases. Given the inconsistent clinical outcomes and high complication rates associated with some stabilization techniques, surgical intervention should be approached with caution and tailored to fragment size and patient demand. However, a universally accepted management pathway has yet to be established.

## Introduction and background

The term "os acromiale" in the international literature refers to a developmental anomaly in which one of the main ossification centers of the acromion fails to fuse with the others, resulting in a fibrocartilaginous connection [1]. The first description of this condition is attributed to the British anatomist John Gregory Smith in 1834, who studied seven shoulder joints from four different cadavers that had sustained trauma to the joint during life. In the anatomical notes he submitted for publication, he described a fracture of the acromial end of the clavicle in the left shoulder of a male specimen extending into the acromioclavicular joint [2]. A similar observation was later reported by the French anatomist Jean Cruveilhier in 1849, who described a linear discontinuity between the acromial epiphysis and the scapular spine in a cadaveric specimen, noting that this finding should not be confused with a fracture [3]. The term os acromiale for this anatomical entity and the recognition that it represents a fibrocartilaginous articulation between adjacent osseous segments were introduced in 1863 by the Austrian anatomist Wenzel Gruber [4]. Subsequently, in 1893, the Irish anatomist Alexander Macalister published an anatomical study of 100 acromia, providing a detailed description of the anatomy and development of the acromion as well as the os acromiale as a developmental anomaly [5]. In the early twentieth century, the first radiographic depiction of this condition was reported in 1914 by the radiologist Lilienfeld, who additionally introduced the distinction between typical and atypical os acromiale, the latter referring to the ectopic presence of ossification centers in the region [6].

Despite being recognized for nearly two centuries, os acromiale remains a topic of clinical interest and debate within orthopedic and musculoskeletal medicine. In most individuals, the acromion develops through the gradual fusion of multiple secondary ossification centers during adolescence and early adulthood. Failure of this fusion results in the persistence of a mobile accessory bone fragment connected to the remainder of the acromion through fibrocartilaginous tissue. Although frequently asymptomatic, this anatomical variation may occasionally be associated with shoulder pain, mechanical impingement, or functional impairment of the shoulder joint.

The reported prevalence of os acromiale in the general population varies considerably among studies. The majority of cases are discovered incidentally during imaging performed for other shoulder pathologies. Nevertheless, in certain individuals, particularly athletes involved in repetitive overhead activities, os acromiale may contribute to symptomatic subacromial impingement, rotator cuff pathology, or chronic shoulder discomfort. As a result, distinguishing between an incidental anatomical variant and a clinically significant condition remains an important challenge for clinicians.

Advances in modern imaging techniques have significantly improved the recognition and evaluation of os acromiale. Conventional radiography, computed tomography (CT), and magnetic resonance imaging (MRI) allow accurate identification of unfused acromial segments and facilitate differentiation from fractures or other pathological entities. Moreover, these imaging modalities provide valuable information regarding associated conditions, including rotator cuff tears or degenerative changes within the subacromial space.

Given the ongoing debate about the clinical relevance of this entity, a thorough understanding of its anatomy, epidemiology, clinical presentation, and management strategies is essential. While os acromiale has been recognized for over a century, the lack of a "gold standard" for surgical management and conflicting data regarding its role in rotator cuff pathology necessitate a contemporary synthesis of the evidence. The purpose of this review is to provide an updated consolidation of current concepts, helping clinicians navigate diagnostic challenges and the evolving landscape of both traditional and arthroscopic treatment options.

## Review

Review methodology

A comprehensive literature search was conducted across the PubMed, Google Scholar, and Scopus databases for articles published from inception to January 2026. The search utilized keywords including "os acromiale", "acromial morphology", and "acromial ossification". Only peer-reviewed articles published in English were included. Reference lists of the retrieved articles were also manually screened to identify additional relevant studies. While the search primarily focused on recent clinical evidence, significant historical anatomical studies from the 19th and early 20th centuries were manually retrieved and included to provide a comprehensive developmental context. Given the rarity of the condition and the predominantly retrospective nature of the available literature, this review synthesizes findings primarily from Level III and IV evidence to provide a comprehensive clinical framework.

Anatomy, embryology, and development of the acromion

Anatomy and Morphological Classification

The acromion is the peripheral anterolateral bony projection of the scapular spine. It serves as an important anatomical landmark, forming the roof of the shoulder girdle with the distal clavicle, which in more than 50% of individuals lies higher than the acromion and partially overlaps it. Macalister’s examination of 100 scapular specimens enabled a detailed description of acromial anatomy. Based on morphological characteristics and the relationships among its constituent parts, acromia were classified as triangular, quadrangular, falciform, or intermediate in shape, with triangular configuration being the most common [5]. Bigliani later proposed a morphological classification based on the angle formed between the acromion and the axis of the scapular spine, describing three types: flat, curved, and hooked acromion [7]. A fourth type, the convex or upturned acromion, was subsequently described by Vanarthos [8]. Although type III acromion was initially thought to be associated with rotator cuff pathology and tears, subsequent studies have produced conflicting results [9].

Ossification Centers

**Figure 1:**
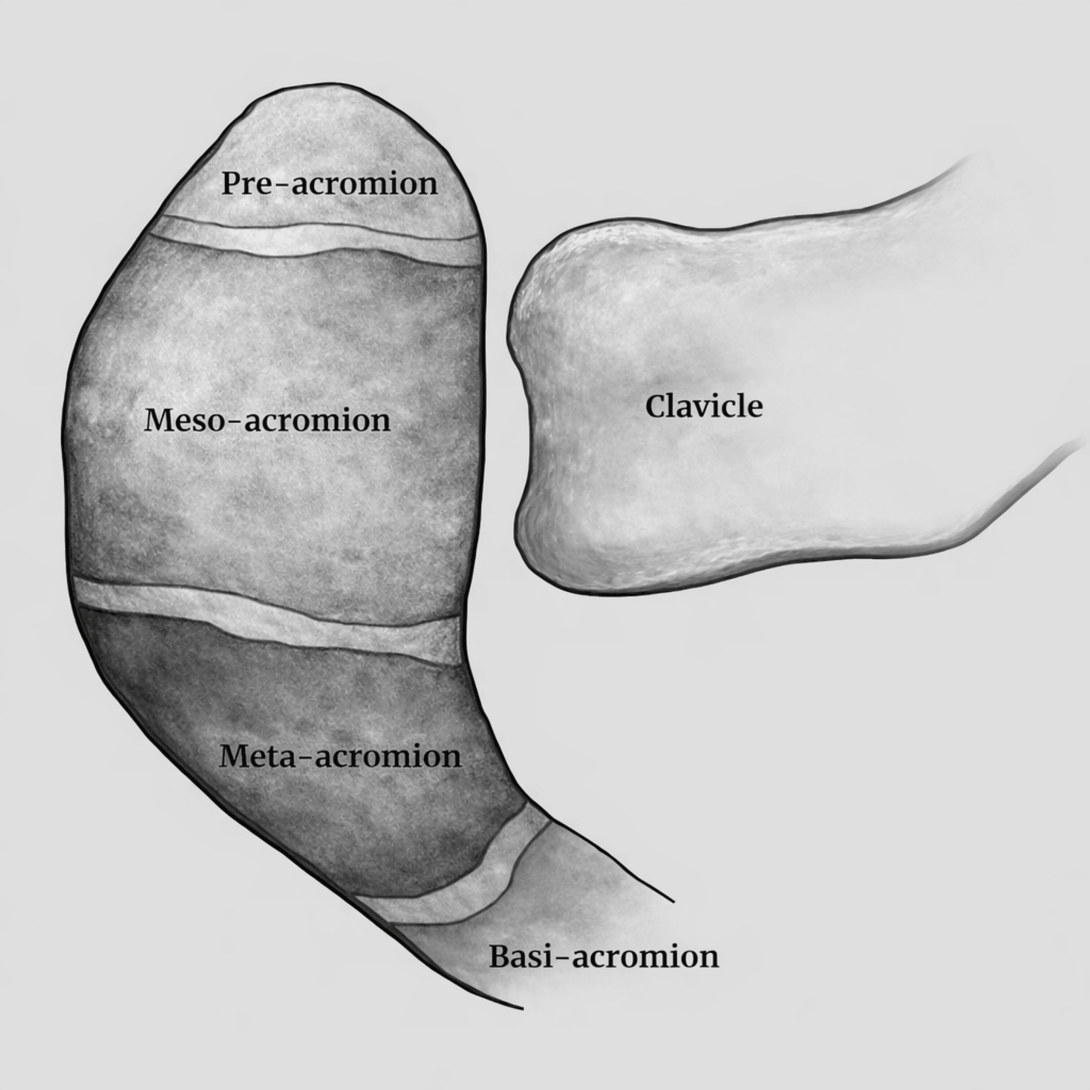
Schematic representation of the acromial process showing the typical locations of synchondroses between the pre-, meso-, meta-, and basi-acromial ossification centers Image Credit: Original illustration created by the author using Sketchpad (sketch.io, Portland, Oregon, USA)

The scapula develops from at least nine ossification centers, three of which contribute to the formation of the acromion. From distal to proximal, these centers are termed the pre-acromion, meso-acromion, and meta-acromion. At the same time, the segment connecting the acromion to the scapular spine is known as the basi-acromion (Figure 1) [10].

Development and Ossification Timeline

The timing and progression of ossification show considerable variability. The cartilaginous model that will eventually form the acromion is already present by the age of two years, although no ossification has yet occurred [5]. In a six-week embryo, the acromion consists primarily of dense mesenchymal tissue, except at its base, where it is continuous with the early cartilaginous body of the scapula [11]. The basi-acromion, which is already formed by the eighth embryonic week, is the first portion to ossify and typically fuses with the scapular spine at approximately 12 years of age. Complete ossification of the acromial segments most commonly occurs between 15 and 18 years but may be delayed until 25 years [12]. Macalister proposed that multiple primordial ossification centers ultimately give rise to the three principal segments of the acromion. The sequence of ossification and fusion among these segments also varies [5]. This detailed chronological sequence of ossification is clinically vital because it provides an objective threshold for distinguishing a symptomatic os acromiale from normal adolescent development.

Imaging Correlates of Acromial Development

The development of the acromion has been further clarified through MRI. Kothary and Rosenberg observed that in children under five years of age, the primary ossification center is already continuous with the scapular spine but does not extend beyond the lateral angle. As development progresses, specifically in the 10-14-year-old age group, the primary ossification center advances anteriorly to form a characteristic inverted, asymmetrical U-shape. Secondary ossification centers typically appear between the ages of 10 and 14 years, with complete fusion most commonly occurring between 14 and 18 years, though it may be delayed in some individuals [13].

Epidemiology

The reported prevalence of os acromiale in the literature is approximately 7% (range 2.7-15%) [4,5,14-20]. Early estimates were based on anatomical dissections and analyses of skeletal collections from archaeological sites and museums, while later studies utilized imaging modalities such as plain radiography, CT, and MRI. Sammarco, after examining 1,198 scapulae from the Hamann-Todd osteological collection in Cleveland, the largest documented collection of skeletons, reported an incidence of 8% [16]. However, Rovesta et al., in their MRI study of 1,042 patients, reported a 3.4% incidence, while Fischer et al., in a larger imaging study of 3,050 MRIs, reported a 1.9% incidence [20,21].

The prevalence appears higher among African and African-American populations (11.1-20%) compared with Caucasian (1.9-12.5%) and Asian populations (0.7%) [21-28]. According to Sammarco's study, os acromiale is more common in men (8.5%) than in women (4.9%); however, a more recent systematic review of 23 studies by Yammine did not identify a statistically significant association between sex and prevalence [16,29].

The condition is bilateral in approximately 33-62% of cases [15-17]. Regarding laterality, results are inconsistent, with some studies suggesting right-sided predominance, others left-sided predominance, and a 2013 review finding no clear preference for either side [20,22,29,30]. Although os acromiale may occur between any of the three main acromial segments and the segment proximal to it, it most frequently occurs between the meso-acromion and meta-acromion [5,14,22].

The significant variability in reported prevalence (2.7% to 15%) likely stems from differences in study methodology and population demographics. Skeletal and cadaveric studies typically report higher rates, as they allow for direct physical inspection of the synchondrosis [4,5,14-16]. In contrast, imaging-based studies, particularly those using plain radiographs, may underestimate the prevalence due to the difficulty of visualizing unfused segments on standard views [17-19]. Furthermore, selection bias in clinical MRI studies, which often analyze symptomatic patients rather than the general population, may further influence the reported epidemiological data [13,20,21].

Etiopathogenesis and clinical presentation

The exact etiology of this anomaly remains unknown. Three principal theories have been proposed. The first attributes its development to mechanical stress during acromial growth, while the second suggests a genetic basis [16,31,32]. The third and more recent theory proposed by Case suggests a combination of both mechanisms [30]. Evidence supporting a genetic contribution includes the approximately threefold higher prevalence among African and African American populations compared with Caucasians, as well as the relatively low prevalence in Asian populations [16,26,30].

Although os acromiale is not rare, with an estimated prevalence of approximately 7% in the general population, it is asymptomatic in most cases [33]. However, several reports, particularly among young athletes, describe symptomatic os acromiale presenting with nonspecific symptoms such as shoulder pain, joint dysfunction, weakness during overhead activities, nocturnal pain, and reduced range of motion. Many of these cases involve athletes engaged in overhead sports such as baseball, tennis, basketball, and swimming [33-38].

While shoulder pain is frequently independent of an existing os acromiale, the condition becomes clinically significant through two primary mechanisms: mechanical instability at the non-union site or a secondary impingement syndrome. In the latter, deltoid contraction during arm elevation causes the mobile fragment to flex inferiorly, compressing the subacromial structures [39]. Furthermore, a previously stable and asymptomatic non-union can be rendered unstable by acute blunt trauma, such as a high-impact collision during contact sports [40].

Earlier studies suggested a high prevalence, approximately half of the cases, of rotator cuff tears and subacromial impingement syndrome in individuals with os acromiale [41-43]. Due to the small sample sizes in the earlier papers, this potential association has been re-examined in later studies with larger sample sizes, which have not demonstrated a statistically significant relationship. In a study by Boehm analyzing 1,000 radiographs of patients with open rotator cuff repair, os acromiale was identified in 6.2% of cases, a rate similar to that observed in the general population [44]. Similarly, Ouellette reported, based on 84 MRI studies of the shoulder, that os acromiale was not a predisposing factor for rotator cuff tears. Still, rotator cuff pathology was more common in patients with os acromiale exhibiting a “step-off” deformity compared with those without such morphological irregularities [45]. However, in a recent multicenter study, Kozono et al. retrospectively analyzed 6,842 shoulder MRIs and found a clear correlation between os acromiale and rotator cuff tear in the Japanese population [28].

These divergent findings likely stem from fundamental differences in study design and population demographics. While Boehm’s analysis used plain radiographs, which may overlook smaller or nondisplaced fragments, Kozono’s multicenter study employed high-resolution MRI, thereby significantly increasing diagnostic sensitivity [28,44]. Furthermore, the discrepancy may be explained by selection bias in earlier, smaller cohorts that focused exclusively on symptomatic surgical patients. In contrast, more recent large-scale data provide a more accurate representation of the broader population [28,41-44].

The clinical challenge remains the differentiation between an incidental radiographic finding and a true symptomatic pathology. While the association with rotator cuff tears remains a subject of ongoing debate, the presence of a "step-off" deformity or MRI-documented instability appears to be a more reliable indicator of clinical significance than the mere presence of the anomaly. Consequently, os acromiale should be viewed not as a primary cause of cuff disease in all patients but rather as a potential mechanical contributor that necessitates symptomatic confirmation through targeted clinical and imaging correlation.

Diagnosis

Symptoms reported in the patient’s history, including shoulder pain, together with tenderness over the acromion and positive impingement signs on clinical examination, are relatively nonspecific. Therefore, more common pathologies with similar clinical presentations should be excluded before considering a diagnosis of os acromiale [46].

The essential radiographic views for diagnosis include the anteroposterior and axillary views, with the axillary view being the most accurate for detection. The anteroposterior view alone may fail to reveal up to half of the cases [17]. Nevertheless, even on the anteroposterior view, the presence of the double-density sign may facilitate diagnosis, particularly in cases where visualization is difficult due to superimposition of the humeral head, even on the axillary view [47]. Because acromial ossification and fusion may occur relatively late, differentiation from an acromial fracture may occasionally be challenging [48].

CT with 3D reconstructions can clearly delineate the os acromiale and provide detailed visualization of the unfused segments [39,49]. However, some authors suggest that these examinations may not always provide significant additional diagnostic information beyond what is available on standard axillary radiographs and classic CT images [49]. After identifying a possible os acromiale and excluding other conditions with similar clinical manifestations, additional imaging modalities such as MRI and SPECT-CT may aid in confirming the diagnosis [20,50-52].

**Figure 2:**
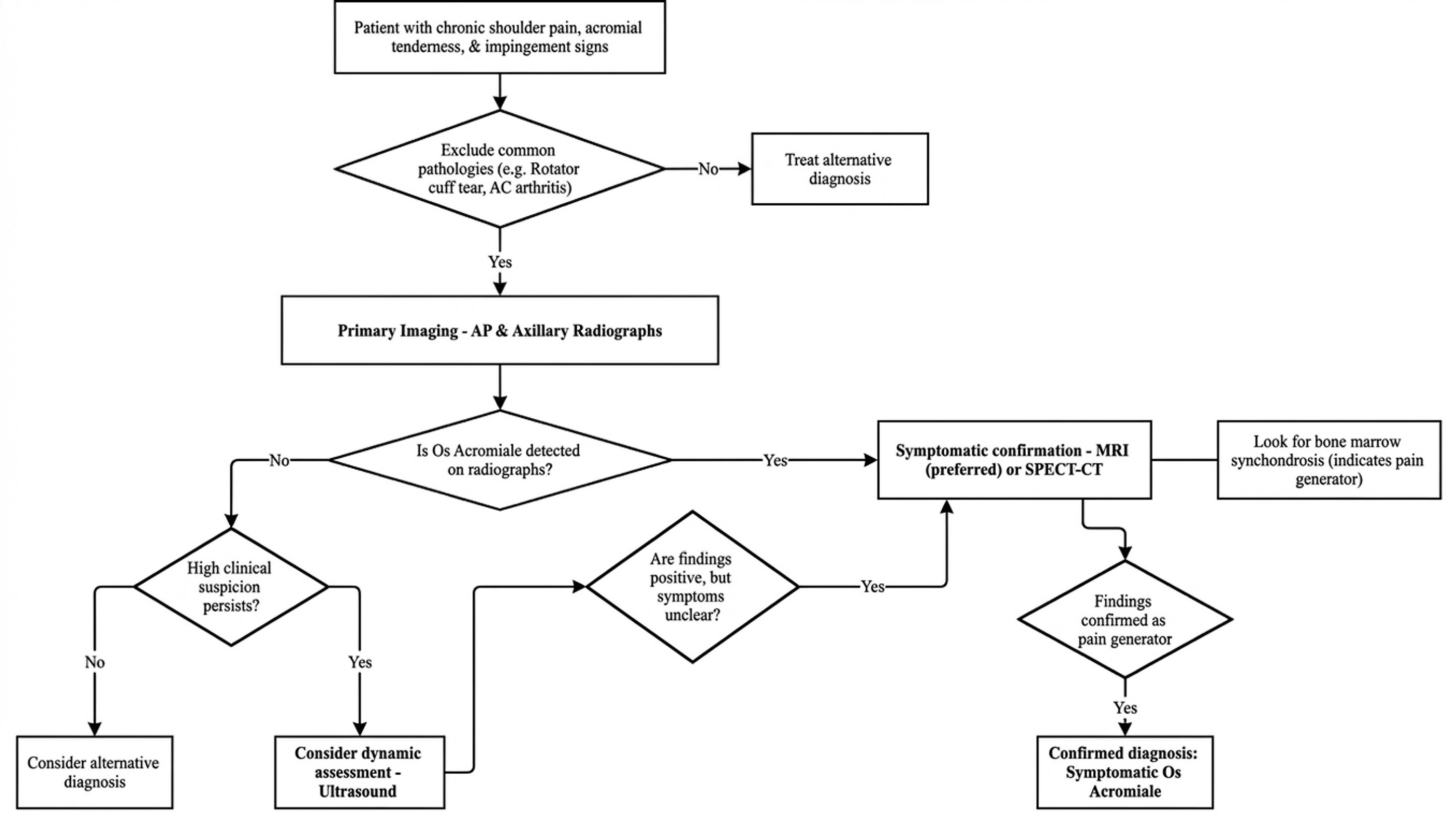
Authors’ proposed diagnostic algorithm for symptomatic os acromiale AP: anteroposterior, AC: acromioclavicular, MRI: magnetic resonance imaging, SPECT-CT: single photon emission computed tomography–computed tomography, CT: computed tomography

On MRI, bone marrow edema between the osseous surfaces may be detected, a finding that is not present during normal acromial development [50]. When plain radiographs and MRI fail to provide definitive findings, SPECT-CT may be the only modality capable of demonstrating os acromiale (Figure 2) [53].

Ultrasound offers a low-cost, non-invasive alternative that enables dynamic assessment and may identify motion at the synchondrosis during shoulder maneuvers. Boehm et al. demonstrated radiologically confirmed os acromiale using ultrasonography in all 25 patients included in their study, suggesting that ultrasound may serve as an alternative imaging modality when the condition is not detected on plain radiographs [54].

Management

Conservative Treatment

Asymptomatic os acromiale does not require treatment [55]. Initial management should be conservative and may include nonsteroidal anti-inflammatory drugs, cryotherapy, rest, and physiotherapy, following treatment principles similar to those used for subacromial impingement syndrome. This is often coupled with significant activity modification, which is particularly vital for athletes or workers involved in repetitive overhead motions that could otherwise exacerbate movement at the unstable segments [33].

Physical therapy remains a cornerstone of this nonoperative approach, centered on strengthening the rotator cuff and scapular stabilizing muscles. By improving overall shoulder biomechanics and restoring range of motion, rehabilitation aims to reduce the mechanical stress placed directly on the unfused site. These protocols typically incorporate stretching and postural correction to further mitigate subacromial impingement [56].

Additionally, local corticosteroid injections into the subacromial space or the fibrocartilaginous interface can provide temporary symptomatic relief. Beyond their therapeutic effect, these injections serve a critical diagnostic purpose by helping clinicians determine if the os acromiale is indeed the primary pain generator in cases of complex shoulder pathology [57].

While many patients, especially those with stable or minimally mobile fragments, achieve satisfactory outcomes through these conservative means, surgical intervention may be required if symptoms persist. Despite the widespread adoption of conservative protocols, the current literature remains constrained by a paucity of high-level evidence, with the quality of evidence limited to retrospective case series and expert consensus [58]. Clinical guidelines generally recommend a trial of nonoperative therapy lasting between six and 12 months [33,39]. Persistent mechanical instability of the synchondrosis and the presence of high-grade concomitant pathology, such as full-thickness rotator cuff tears, represent primary predictors of conservative failure. In such instances, definitive surgical stabilization or excision is required for long-term functional restoration [33].

Surgical Treatment

When evaluating surgical success, a systematic review by Harris et al. provides a comprehensive synthesis of the literature [58]. Various surgical techniques have been described, including open or arthroscopic excision of the osseous fragment, acromial resection, open reduction and internal fixation with or without bone grafting, arthroscopically assisted reduction and fixation, and arthroscopic subacromial decompression. While patient satisfaction is generally high across different techniques, the lack of prospective randomized trials remains a significant limitation in the field (Table 1).

**Table 1:** Comparative summary of surgical techniques for OS acromiale: indications, technical considerations, and clinical outcomes

Technique	Approach/method	Primary indication	Key technical points	Clinical results and complications
Excision	Open	Symptomatic small pre-acromion fragment	Requires separation and careful reattachment of the deltoid origin to prevent functional loss	Improved results for small fragments; poor outcomes for large fragments or inadequate deltoid repair (weakness, pain)
	Arthroscopic		Minimal or no disruption of deltoid attachment and periosteal sleeve	Improved clinical results; currently lacks direct comparative data against open excision
Acromioplasty	Open	Symptomatic stable meso-acromion fragment	Reduces dynamic bony impingement on the rotator cuff and bursa; requires meticulous deltoid repair	Equivalent results to fixation/excision for stable fragments; poor results if used for unstable, mobile fragments
	Arthroscopic		Reduces dynamic impingement while avoiding disruption of the deltoid origin	Improved clinical results; currently lacks direct comparative data against open acromioplasty
Internal fixation (+/- grafting)	K-wire technique (+/- tension band)	Unstable meso-acromion fragment	Stabilizes the fragment against the deltoid pull; less rigid than screws	Risks include hardware pullout, skin irritation from prominent hardware, and persistent pain
					Screw technique (+/- tension band)	Biomechanically more powerful and rigid construct than K-wires. Arthroscopically-assisted fixation provides blood supply preservation and minimizes deltoid injury, but remains challenging	Higher radiographic healing rates and better clinical outcomes than K-wires; reduced need for hardware removal. Better clinical outcome for healed fragments than those that result in non-union (technique independent)

Open or Arthroscopic Resection of Fragment

The surgical management of os acromiale through resection, whether via open or arthroscopic techniques, remains a subject of significant clinical debate, primarily due to the risk of compromising the deltoid muscle’s structural integrity. While general recommendations suggest that small acromial fragments are candidates for excision and larger fragments are better suited for fusion, the clinical outcomes of open resection have historically been inconsistent [59,60]. The primary concern with open excision is the potential for postoperative weakness and functional loss caused by the detachment and subsequent repair of the deltoid [61].

Evidence regarding open excision results varies across several key studies. Mudge et al. reported on a series of patients with concurrent rotator cuff tears and os acromiale. In contrast, four of six patients achieved excellent results following fragment excision and deltoid reconstruction; two suffered poor outcomes [59]. Similarly, Warner et al. reported success with pre-acromion excision but observed poor results in meso-acromion cases, characterized by lingering pain and weakness [62]. In a larger study by Boehm et al. involving 33 patients, researchers compared excision, acromioplasty, and fusion following rotator cuff repair. Interestingly, they found no statistically significant differences in functional scores across all groups, including those with unsuccessful fusions. This suggests that clinical improvement can occur even in the absence of radiological union. Their findings indicate that small mobile fragments are best resected, while large stable fragments may only require acromioplasty, and large unstable fragments should ideally be fused [63].

However, the historical perspective on radical resection is cautionary. A study by Armengol et al., which reviewed 41 patients, failed to yield satisfactory results with excision techniques, in contrast to Mudge’s earlier small-series success [60]. This skepticism is heavily supported by the principles of Neer, who warned that radical acromionectomy, defined as the removal of more than 80% of the acromion, severely defunctions the deltoid lever arm. In a series of 30 such cases, 90% of patients suffered from persistent pain and an inability to lift the affected limb above 90 degrees [61].

In recent years, arthroscopic excision has emerged as a potentially superior alternative to open surgery. The primary benefit of the arthroscopic approach is the preservation of the periosteal and deltoid attachments, which minimizes the risk of muscle dysfunction. Research by Campbell et al. found no decrease in deltoid strength or function compared to the contralateral arm, regardless of whether a rotator cuff repair was performed simultaneously [64]. Further case reports, such as those by Kawaguchi et al., support the efficacy of arthroscopic resection for unstable meso-acromion fragments, achieving successful impingement relief without residual deltoid deficits [65]. Collectively, the data suggest that while fragment size and stability dictate the surgical path, preserving the deltoid lever arm is the most critical factor for a successful functional outcome. Nevertheless, no studies have directly compared the surgical outcomes of open versus arthroscopic excision of an os acromiale fragment, according to Viner et al.'s systematic review [66].

Open or Arthroscopic Assisted Reduction and Internal Fixation

The surgical management of an unstable and painful os acromiale relies heavily on internal fixation to stabilize the fragment and neutralize the pulling forces of the deltoid muscle. Case series evaluating open reduction and internal fixation techniques, such as tension band wiring, suturing techniques, or cannulated screws with or without bone grafting, have generally demonstrated favorable outcomes [62,67-69]. While fusion is the primary goal, it is notoriously difficult to achieve. To improve success rates, many surgeons advocate for a transacromial surgical approach, which preserves the terminal branches of the thoracoacromial artery and maintains a healthier physiological environment for bone healing [68,70]. Additionally, the use of bone grafts, whether harvested locally from the non-union site or from the iliac crest, is common. A recent comparative study by Atinga et al. suggested that local bone grafting is just as effective as iliac crest grafting, achieving clinical and radiological fusion in as little as 3 months without the added morbidity of a secondary donor site [71].

Technical precision during the procedure is critical. Surgeons typically excise the sclerotic edges of the pseudarthrosis using marginal dorsal wedge cuts. By fixing the anterior portion of the acromion in a slightly upward tilt, the subacromial space naturally increases, often making a formal acromioplasty unnecessary [62]. In terms of hardware, while traditional K-wires and tension band wiring have been used, they are frequently associated with non-union and hardware-related discomfort. Consequently, the combination of cannulated screws with a tension band construct has become a preferred standard. Biomechanical research supports this "hybrid" approach, demonstrating that screws augmented with tension band wiring have a 39% higher load-to-failure strength than screws used in isolation [72]. Viner et al., in their review, indicate that for meso os-acromiale, internal fixation with screws, compared with K-wire fixation, has a higher rate of radiographic union, improved patient outcomes, and a reduced need for postsurgical hardware removal [66]. The use of cannulated compression screws, in an anteroposterior direction, as an isolated construct has also been described by Ryu et al. [69].

Advancements in arthroscopic techniques offer a less invasive alternative for internal fixation. Arthroscopically-assisted fixation aims to preserve the blood supply and minimize deltoid injury by avoiding muscle detachment entirely. Some protocols involve debriding the non-union site through a lateral portal and using biodegradable screws to compress the fragments. While these techniques yield high patient satisfaction and excellent cosmetic results, they do present challenges; specifically, the biomechanical strength of absorbable hardware is still being studied, and the limited maneuverability makes it difficult to achieve the "tilted upward" position often required to decompress the subacromial space without additional bone removal [73].

Open or Arthroscopic Acromioplasty

Clinical outcomes for treating meso-os acromiale with acromioplasty or partial resection show consistent improvements in pain and function, though success rates vary across studies. This technique is particularly effective for stable os acromiale, where it reduces dynamic bony impingement of the rotator cuff and subacromial bursa against the acromial undersurface.

Boehm et al. found that open acromioplasty significantly improved pain components in the Constant score (from 4.6 to 12.2), yielding an 80% satisfaction rate. Compared with age- and sex-matched cohorts undergoing more invasive internal fixation or open excision, results were comparable, with all groups achieving a similar overall Constant score [63]. Further evidence from Abboud et al. evaluated 11 patients undergoing either arthroscopic or open Neer acromioplasty, reporting a 63.6% satisfaction rate [74].

The efficacy of arthroscopic techniques is further supported by Johnston et al., who utilized partial resection and acromioplasty for meso-type fragments, with pain reduction being the most significant outcome [57]. However, as highlighted by the systematic review by Harris et al., the success of this approach is highly dependent on fragment stability. While results with stable fragments are equivalent to excision or fixation, acromioplasty for unstable, mobile fragments may not sufficiently reduce dynamic impingement, potentially leading to inferior clinical results. Furthermore, a critical surgical consideration is preserving the deltoid origin; arthroscopic techniques should avoid disrupting the attachment, whereas open procedures require meticulous repair to maintain functional integrity [58]. Collectively, these findings suggest that for a stable meso-os acromiale, addressing impingement through acromioplasty is a highly effective, less invasive alternative to fragment excision or fusion.

## Conclusions

While os acromiale is a common developmental anomaly, its transition from an incidental finding to a clinical driver of shoulder pathology remains a subject of significant debate. Diagnosis requires a nuanced approach, particularly in patients under 25, to differentiate physiological variants from true symptomatic non-unions. Central to current clinical controversy is the lack of consensus on the optimal imaging algorithm. While axial radiographs are fundamental, the comparative diagnostic weight of MRI versus SPECT-CT in identifying the active pain generator remains to be fully defined.

Furthermore, management strategies continue to lack a "gold standard" protocol. While open reduction and internal fixation is the most established technique, the potential superiority of emerging arthroscopic methods is an area of active investigation. Ultimately, the absence of high-level comparative studies necessitates a highly individualized management plan that balances the patient’s functional requirements against the technical challenges and variable union rates associated with current surgical interventions.
